# Review of the prevalence of foodborne pathogens in milk and dairy products in Ethiopia

**DOI:** 10.1016/j.idairyj.2020.104762

**Published:** 2020-10

**Authors:** Abdi Keba, M. Laura Rolon, Aynadis Tamene, Kindinew Dessie, Jessie Vipham, Jasna Kovac, Ashagrie Zewdu

**Affiliations:** a Ethiopian Institute of Agricultural Research, Holeta Agricultural Centre, PO Box 036, Addis Ababa, Ethiopia; b Department of Food Science, The Pennsylvania State University, 202 Rodney A. Erickson Food Science Building, University Park, PA 16802, USA; c Centre for Food Science and Nutrition, Addis Ababa University, New Graduate Building, College of Natural Sciences, PO Box 1176, Addis Ababa, Ethiopia; d Department of Plant Science, University of Aksum, PO Box 314, Aksum, Ethiopia; e Department of Animal Science and Industry, Kansas State University, 108 Waters Hall, Manhattan, KS 66506, USA

## Abstract

Food safety is a significant barrier to social and economic development throughout the world, particularly in developing countries. Here, we reviewed the prevalence of major bacterial foodborne pathogens (*Salmonella* spp., *Listeria monocytogenes*, *Escherichia coli* O157:H7 and *Campylobacter* spp.) in the rapidly growing Ethiopian dairy supply-chain. We identified 15, 9, 5 and 0 studies that had reported the prevalence of *Salmonella* spp., *L. monocytogenes, E. coli* O157:H7, and *Campylobacter* spp. in dairy foods, respectively. The studies reviewed reported a median prevalence of *Salmonella, L. monocytogenes,* and *E. coli* O157:H7 of 6, 9 and 10%, respectively, in raw cow milk in Ethiopia, indicating a concerning occurrence of bacterial foodborne pathogens in raw milk. Implementation of good hygiene and production practices and assessment of interventions targeting the reduction of contamination in the dairy supply chain is needed to inform coordinated efforts focused on improvement of dairy food safety in Ethiopia.

## 1 Introduction

Food safety is a significant barrier to the socio-economic development of nations across the globe. The World Health Organisation (WHO) estimated that in 2010, foodborne pathogens were responsible for 600,652,361 illness cases and 418,608 deaths worldwide (Havelaar et al., 2015). Africa was reported to have the highest burden of foodborne diseases per capita, with a median of 2,455 foodborne Disability Adjusted Life Years (DALYs) per 100,000 inhabitants (WHO, 2015). Of these, 26.6% were attributed to *Salmonella* spp., 11.2% to enteropathogenic *Escherichia coli*, 8.6% to enterotoxigenic *E. coli*, 0.08% to *Listeria monocytogenes*, 5.7% to *Campylobacter* spp., and 0.004% to Shiga-toxin producing *E. coli* (Havelaar et al., 2015, WHO, 2015). In Ethiopia, diarrhoea was reported as the second contributor to the total burden of all disease types and the leading cause of premature death (Misganaw et al., 2017). Diarrhea has also been associated with growth stunting in children, which can have long-term development consequences (Rogawski et al., 2018).

Adequately managed and enforced Good Manufacturing Practices (GMP) and food safety monitoring and surveillance are important components of modern food supply systems that play a critical role in the control of foodborne pathogens (ICMSF, 2002). However, food systems in Africa are frequently uncoordinated and poorly regulated, resulting in compromised food safety and protection of public health from foodborne illness (FAO, 2005). Furthermore, weak food safety systems limit opportunities for economic growth through international trade of domestically produced food products (FAO, 2005). In Ethiopia, the food safety regulation is fragmented between several agencies, including the Ministry of Health, the Ministry of Agriculture, Ministry of Trade and Industry, The Quality and Standards Institution, and local governments and municipalities (Ayalew, 2013, Birke and Zawide, 2019). However, there is a lack of regulation that would dictate the function of each government body and the nature of cooperation among them (Temesgen, 2015). Due to the lack of food safety regulation, and the existing limited analytical capabilities at national and regional levels, Ethiopia has yet to develop a foodborne diseases surveillance system coordinated at a national level (Birke & Zawide, 2019).

Due to the lack of surveillance, there are also no published data on the incidence of foodborne illness in Ethiopia, further emphasising the need for the implementation of surveillance systems. Such system could provide systematic baseline data on the prevalence of major foodborne pathogens in the supply chain and the prevalence of foodborne illness, to inform prioritisation of pathogen control intervention strategies. Moreover, although challenging, implementation of inspection and quality control services for foods of animal origin could provide benefits to the public in terms of safeguarding the public from zoonotic diseases (FAO, 2005). As a result of the absence of the food safety surveillance system, there is a severe lack of baseline epidemiological data, which disables prioritisation and informed implementation of policies focused on relieving the burden of public health costs due to foodborne diseases. This review of published peer-reviewed literature reporting the prevalence of bacterial foodborne pathogens in the rapidly growing Ethiopian dairy supply chain was carried out to provide a summary of the bacterial foodborne pathogen prevalence. The data reported here may be used to inform future food safety interventions aimed at reduction of the incidence of foodborne illness due to exposure to pathogens through consumption of dairy foods in Ethiopia.

## 2 Approach

A comprehensive literature review was performed by searching databases of scientific literature reporting the prevalence of *Salmonella* spp., *L. monocytogenes*, *E. coli* O157:H7, and *Campylobacter* spp. in milk and dairy products in Ethiopia. In addition, articles that reported the prevalence of commonly used microbial indices for these pathogens, such as *Listeria* spp. for *L. monocytogenes* were included in the review. The databases PubMed, Web of Science, and African Journals Online were searched using the keywords “Ethiopia” AND (“dairy” OR “milk”) AND (“*Listeria*” OR “*Salmonella*” OR “*E. coli* O157” OR “*Campylobacter*”) AND/OR “prevalence” for articles published between 2004 and 2020. The results of each search were filtered based on the relevance of the title and abstract. Only peer-reviewed studies that reported prevalence of bacterial foodborne pathogens relevant to milk and dairy products in Ethiopia were included in the summaries of pathogen prevalence reported in this review. In addition, for each selected publication, the reference section was examined to identify additional relevant publications.

Each article was read by two independent reviewers to identify the following information of interest: (i) the place and time the study was conducted, (ii) the experimental design and needed sample size estimation, (ii) the methods used to detect each pathogen and (iv) the prevalence of each pathogen in each dairy product type. Summary statistics were calculated using the package psych (version 1.8.12) (Revelle, 2019) and boxplot figures were created using the package ggplot2 (version 3.2.1) (Wickham, 2009) in R (version 3.6.1) (R Core Team, 2019) to summarise the mean and distribution of results obtained from included peer-reviewed publications. The geographical location of each study was mapped using the R package tmap (version 2.3–1) (Tennekes, 2018).

## 3 Discussion

### 3.1 Ethiopia's dairy supply chain

**Fig. 1:**
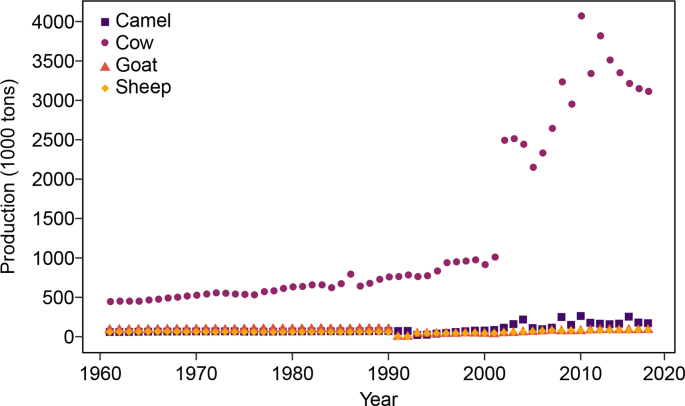
Milk production in Ethiopia, by animal species (FAO, 2019).

Ethiopia's dairy production experienced a remarkable growth between 2000 and 2010, and has stabilised at 3,100,000 tons of milk per year (Fig. 1), according to the data reported by the Food and Agriculture Organisation (FAO) of the United Nations (FAO, 2019). Milk production systems in Ethiopia are generally classified into three categories based on geographical location: rural, peri-urban and urban. In the rural system, milk is mainly produced for household consumption, and leftovers are sold in local informal markets. Rural systems can be further subdivided into production by pastoralists (limited to the lowlands), agro-pastoralists and mixed crop-livestock producers (Getabalew, Alemneh, & Akeberegn, 2019). The rural system has been reported to account for 98% of the country's milk production (Gebremichael et al., 2019, Tadesse, 2018). The minority of milk was reported to be produced in peri-urban systems that include smallholder and commercial farms in the suburban areas near cities that have available grazing land (Getabalew et al., 2019). Lastly, a small proportion of milk is produced in urban areas that are limited to farms close to cities, and that have no access to grazing lands (Gezu and Tadesse, 2018). According to Tadesse (2018), the urban milk system produces 34.65 million litres of milk annually, which accounts for approximately 1% of the country's total milk production, considering the country's total milk production reported by FAO (FAO, 2019). Ethiopia has the potential to expand its dairy production, due to the suitable geographical and climate conditions, in particular in the highlands (Gezu and Tadesse, 2018). According to the FAO, Ethiopia has the eleventh largest cattle inventory in the world, and the largest cattle inventory (including both dairy and beef cattle) in Africa, which demonstrates it suitability for growing the country's livestock sector (FAO, 2019). The main identified limitations for the growth of the dairy sector include the low yield of milk per animal (currently 1.54 L per day), control of infectious and parasitic diseases of cattle, access to grazing land, and poor milk handling practices that lead to poor microbiological milk quality (Getabalew et al., 2019). Improvement of good hygiene and manufacturing practices and milk microbiological quality could potentially enhance the growth of the dairy supply chain by enhancing not only milk safety, but also its quality and therefore shelf life and accessibility.

### 3.2 Consumption of raw milk and associated health risks

Consumption of milk in Ethiopia was reported to be between 17 and 19 L per capita, which is lower than the regional average (e.g., 90 L per capita in Kenya and 50 L per capita in Uganda) (Gebremichael et al., 2019, Girma and Abebe, 2018). However, milk demand is increasing due to migration to urban areas (Gezu and Tadesse, 2018). Ethiopian consumers generally prefer unprocessed (i.e., raw) fluid milk due to its flavour, availability and price (Amenu et al., 2019, Girma and Abebe, 2018), however, it is not known what is the exact proportion of milk that is consumed raw.

A study on perception of milk processing and consumption practices reported that pastoralists in southern Ethiopia do not boil milk before consumption because they perceive this process as detrimental, in terms of nutritional quality (Amenu et al., 2019). However, the pastoralist population may not be representative of the Ethiopian population as a whole. Given that milk contains a wide range of nutrients and is highly susceptible to microbial spoilage, the conditions in which milk is collected, transported, and stored will affect not only its quality and shelf stability, but also microbial safety (Walstra, Wouters, & Geurts, 2006).

Milking carried out in unhygienic environments increases the likelihood of milk contamination by zoonotic pathogens, the level of which can subsequently increase due to pathogen growth when milk is stored at ambient temperatures (Bekuma, 2018, Tegegne and Tesfaye, 2017, Tigabu et al., 2015). Therefore, consumption of raw milk without pasteurisation results in an increased risk for exposure to bacterial foodborne pathogens, such as *Campylobacter* spp., *L. monocytogenes*, *Salmonella* ssp., and *E. coli* O157:H7, as well as other zoonotic agents such as *Brucella* spp. and *Coxiella burnetii* (Walstra et al., 2006). A proportion of milk produced in Ethiopia is processed into dairy products, such as fermented milk (ergo), curd milk with partial removal of whey (ititu), cottage cheese-like product (ayib), butter and cream (used as whipped cream-based filling for cakes) (Amenu et al., 2019).

Through our literature review, we did not identify any reliable data reporting the proportion of milk used for production of individual products and whether the milk processed into these products is pasteurised. However, anecdotally, the artisanal production of dairy products typically uses raw milk. The microbial safety of these products is highly variable, as determined by bacterial foodborne pathogen occurrence; which poses a health risk to consumers. In the following sections, we report a summary of the literature review on the prevalence of four major bacterial foodborne pathogens (Abera et al., 2016, Abunna et al., 2017; Abunna et al., 2018a, Abunna et al., 2018b, Abunna et al., 2018c, Addis et al., 2011, Adugna et al., 2015, Adugna et al., 2013, Banti, 2018, Bedasa et al., 2018, Desta, 2013, Disassa et al., 2017, Ejo et al., 2016, Fisseha, 2017, Garedew et al., 2015, Gebretsadik et al., 2011, Girma and Abebe, 2018, Mekuria and Beyene, 2014, Mengesha et al., 2009, Molla et al., 2004, Mossie and Dires, 2016, Muhammed et al., 2013, Mulaw, 2017, Reta et al., 2016, Seyoum et al., 2015; Tadesse and Dabassa, 2012, Tesfaw et al., 2013, Tesfay et al., 2013).

### 3.3 Reported prevalence of *Salmonella* spp. in milk and dairy products

*Salmonella* is the leading cause of foodborne illness worldwide (Havelaar et al., 2015). *Salmonella* spp. are Gram-negative, rod-shaped, facultative anaerobic organisms known to cause salmonellosis in humans. Within the genus *Salmonella*, *Salmonella enterica* is the species which can be further classified into six subspecies, with *S. enterica* subspecies *enterica* being responsible for 99% of the infections in humans and animals (Jajere, 2019). *Salmonella* outbreaks have been linked to poultry and beef products, fruit, produce and ready-to-eat meals (CDC, 2019). Dairy products, including raw milk and soft unpasteurised cheese have been identified as sources of *Salmonella* during past outbreak investigations (Maguire et al., 1992, Olsen et al., 2004, Ryan et al., 1987). The most common transmission source for human infections with *Salmonella* is the consumption of foods that have been cross-contaminated with animal faeces or matter from natural environment (Jajere, 2019).

**Fig. 2:**
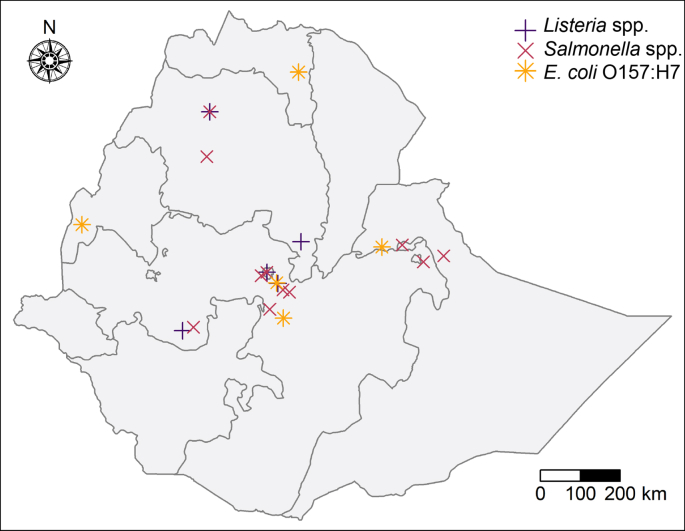
Geographical locations at which the reviewed studies investigated the prevalence of foodborne pathogens in Ethiopian milk and dairy products.

In Ethiopia, the prevalence of *Salmonella* has mostly been studied in slaughterhouses and abattoirs (Ejeta et al., 2004, Molla and Mesfin, 2003, Molla et al., 2006). A meta-analysis on the prevalence of *Salmonella* spp. in raw animal products in Ethiopia included only three studies that collected and analysed milk samples (Tadesse & Gebremedhin, 2015). In this review, fifteen peer-reviewed publications were identified and reviewed to summarise the prevalence of *Salmonella* spp. in Ethiopian milk and dairy products (Abate et al., 2013, Abera et al., 2016, Abunna et al., 2017, Abunna et al., 2018a, Abunna et al., 2018b, Addis et al., 2011, Adugna et al., 2013, Banti, 2018, Ejo et al., 2016, Mossie and Dires, 2016, Mulaw, 2017, Reta et al., 2016, Tadesse and Dabassa, 2012, Tesfaw et al., 2013, Tesfay et al., 2013). In addition, two non-peer reviewed theses (Dailey, 2011, Hunduma, 2018) were identified as relevant but were not included in further analysis and data summary. Table 1 reports the experimental design and methods for detection and identification of *Salmonella* spp. in milk and dairy products used in the reviewed studies. Fig. 2 shows the geographical areas in which reviewed studies were carried out. Three studies were conducted in Addis Ababa, of which two collected samples from urban farms (Addis et al., 2011, Banti, 2018) and one purchased samples at retail stores (Tesfaw et al., 2013). Seven studies were conducted in the Oromia region, five of which were conducted in towns less than 100 km away from Addis Ababa (Abate et al., 2013, Abunna et al., 2017, Abunna et al., 2018a, Mossie and Dires, 2016) and two were conducted in towns over 100 km from Addis Ababa (Abunna et al., 2018b, Tadesse and Dabassa, 2012). Two studies were conducted in Amhara region (Ejo et al., 2016, Mulaw, 2017), three in Somali region (Abera et al., 2016, Adugna et al., 2013, Reta et al., 2016) and two in Harar (Adugna et al., 2013, Tesfay et al., 2013). Twelve studies reported employing a cross-sectional study design (Abera et al., 2016, Abunna et al., 2017, Abunna et al., 2018a, Abunna et al., 2018b, Banti, 2018, Ejo et al., 2016, Mossie and Dires, 2016, Mulaw, 2017, Reta et al., 2016, Tesfaw et al., 2013), of which seven reported a sample size calculation based on the prevalence estimates between 2.1% and 50% (Abunna et al., 2018b, Abunna et al., 2017, Addis et al., 2011, Banti, 2018, Mossie and Dires, 2016, Mulaw, 2017, Tesfaw et al., 2013) (see Table 1).

**Table 1:** Experimental design and locations of reviewed studies that reported the prevalence of *Salmonella* spp. in dairy products in Ethiopia.^a^

Reference	Region	Town	Dates	Place of sampling	Study design	Sample size calculation	Detection method	Confirmation method
Addis et al. (2011)	Addis Ababa	Addis Ababa	Feb 2010 to May 2010	Collected from dairy farms	Not stated	Sample size determined based on P = 7.1%, d = 5%, confidence = 95%	Pre-enrichment in peptone water, enrichment in Selenite cysteine and RV broths, isolation in XLD	Biochemical tests: TSI, Urea agar, LIA, VP, methyl red and indole test
Tadesse and Dabassa (2012)	Oromia	Kersa, Jimma Zone	Dec 2010 to Jun 2011	Collected from farmers and milk collection centres	Random sampling	Not stated	Enrichment in RV soy peptone, isolation on BGA and XLD agar	Biochemical tests: LIA, TSI, Urea agar, Simmons citrate agar and SIM. Agglutination tests
Abate et al. (2013)	Oromia	Sebeta	Not stated	Collected form household lactating healthy exotic cows	Not stated	Not stated	ISO-6579:2002	Biochemical tests: indole, methyl red, VP, lysine decarboxylation, hydrolysis and production of hydrogen sulphide
Tesfaw et al. (2013)	Addis Ababa	Addis Ababa	Not stated	Purchased at supermarkets	Cross-sectional	Random sampling. Sample size determined based on P = 50%, d = 5%, confidence = 95%	ISO-6579:2002	Biochemical tests: ISO 6579:2002
Tesfay et al. (2013)	Dire Dawa	Dire Dawa	Not stated	Collected from bulk milk containers and milk vendors	Not stated	Not stated	Pre-enrichment in peptone water, enrichment in Selenite cysteine broth, isolation in XLD	Not performed
Adugna et al. (2013)	Somali and Dire Dawa	Erer and Dire Dawa	Not stated	Collected from producers' households and markets	Not stated	Not stated	Pre-enrichment in peptone water, enrichment in selenite cysteine broth and isolation in XLD	Not performed
Abera et al. (2016)	Somali	Bebile and Gursum districts	Not stated	Collected from camel herds in the area from the udders, milking buckets and the market	Cross-sectional	Not stated	Coliform count plates in VRBA plates and isolation on SS plates	TSI
Ejo et al. (2016)	Amhara	Gondar	Feb 2014 to Dec 2015	Purchased from restaurants, hotels, cafeterias, pastry and retail shops	Cross-sectional	Random sampling	ISO-6579:2002	Biochemical tests: TSI, urea agar, LIA, VP, methyl red, Simmons citrate and indole test
Mossie and Dires (2016)	Oromia	Debre Zeit	Dec 2013 to Apr 2014	Collected from bulk tank milk from household/small holders, supermarket and large dairies	Cross-sectional	Sample size determined based on P = 2.1%, d = 5%, confidence = 95%	ISO-6579:2002	Biochemical tests: indole, methyl red, VP, urea broth, LIA and TSI
Reta et al. (2016)	Somali	Jigjiga	Mar 2013 to Jan 2014	Collected from individual farmers, milk collectors and milk vendors	Cross-sectional	Not stated	Pre-enrichment in peptone water, enrichment in Selenite cysteine broth, isolation in XLD	Biochemical tests: LIA, TSI, urea agar, VP broth, methyl red and indole test
Abunna et al. (2017)	Oromia	Modjo	Jan 2016 to Apr 2016	Collected at dairy farms from the udders of cows and the bulk tank	Cross-sectional	Random sampling. Sample size determined based on P = 28.6%, d = 5%, confidence = 95%	ISO-6579:2002	Biochemical tests: indole, methyl red, VP, urea broth and TSI
Mulaw & Town (2017)	Amhara	Bahir Dar	Nov 2012 to Jun 2013	Collected at dairy farms	Cross-sectional	Sample size determined based on P = 50%, d = 5%, confidence = 95%	Pre-enrichment in peptone water, enrichment in Selenite cysteine broth, isolation in XLD	Biochemical tests:: Kliger iron agar, urea agar, SIM, lysine deoxycholate agar and Simmons citrate agar
Abunna et al. (2018b)	Oromia	Meki	Jan 2016–Apr 2016	Collected at dairy farms from the udders of cows and the milking bucket	Cross-sectional	Random sampling. Sample size determined based on P = 10.7%, d = 5%, confidence = 95%	ISO-6579:2002	Biochemical tests: ISO 6579:2002
Abunna et al. (2018a)	Oromia	Adama	Feb 2014 to Apr 2014	Collected at dairy farms from the bulk tank	Cross-sectional	Not stated	ISO-6579:2002	Biochemical tests: ISO 6579:2002
Banti (2018)	Addis Ababa	Addis Ababa	Jan 2017 to May 2017	Collected at dairy farms from the udders of cows	Cross-sectional	Random sampling. Sample size determined based on P = 7.1%, d = 5%, confidence = 95%	Pre-enrichment in peptone water, enrichment in Selenite cysteine and RV broths, isolation in SS agar and XLD	Biochemical tests: TSI, urea agar, indole, methyl red, VP broth and citrate test

a Abbreviations: d, precision; P, prevalence; BGA, brilliant green agar; LIA, lysine indole agar; RV, Rappaport-Vassiliadis broth; SIM, sulphide indole motility medium; SS, *Salmonella-Shigella* agar; TSI, triple sugar iron; TT, tetrathionate broth; VP, Voger Proskauer medium; VRBA, violet red bile agar; XLD, xylose lysine deoxycholate agar.

Seven peer-reviewed publications used ISO 6579:2002 (ISO, 2002) as a standard method for detection of *Salmonella* spp. in food products (Abate et al., 2013, Abunna et al., 2017, Abunna et al., 2018a, Abunna et al., 2018b, Ejo et al., 2016, Mossie and Dires, 2016, Tesfaw et al., 2013). Common biochemical tests, described as part of ISO standard method, measured the ability of presumptive *Salmonella* spp. isolates to ferment glucose, sucrose and lactose, as well as its ability to produce hydrogen sulphide (TSI), to decarboxylate lysine (LIA) and to hydrolyse urea into ammonia. In addition, optional tests for the detection of β-galactosidase activity and indole reaction can be added to the protocol. Six studies used a modification of the ISO protocol that utilised selenite cysteine broth as secondary enrichment medium (Addis et al., 2011, Adugna et al., 2013, Banti, 2018, Mulaw, 2017, Reta et al., 2016, Tesfay et al., 2013). Two studies (Abera et al., 2016, Tesfay et al., 2013) did not perform all biochemical tests recommended by the ISO method to confirm the identity of putative *Salmonella* spp. isolates.

**Fig. 3:**
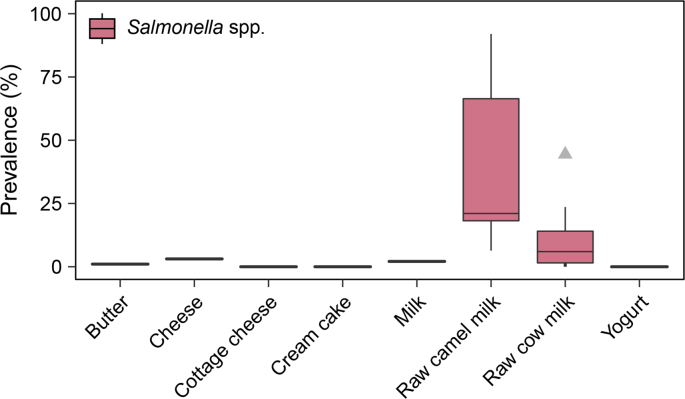
Prevalence of *Salmonella* spp. in milk and dairy products collected in Ethiopia. N = number of studies; n = number of samples: butter (N = 1, n = 96), cheese (N = 1, n = 96), cottage cheese (N = 1, n = 20), cream cake (N = 1, n = 50), milk (N = 1, n = 96), raw camel milk (N = 2, n = 150), raw cow milk (N = 12, n = 1393), yogurt (N = 1, n = 96).

Reviewed studies tested milk of different animal species. Two studies collected samples of raw camel milk (n = 150) and reported a median prevalence of *Salmonella* spp. of 21%, with prevalence ranging from 6 to 92% (Abera et al., 2016, Adugna et al., 2013) (Fig. 3, Supplementary material Table S1). Camel milk is traditionally produced and consumed by pastoralist communities in arid and semi-arid regions of eastern Africa, including Ethiopia, as a source of nutrition, particularly for children (Adugna et al., 2013, Gebremichael et al., 2019). Previous studies on the quality of raw camel milk in Ethiopia have indicated high bacterial and coliform counts in raw milk collected from the udder, and from the market, and recommended focussing on improvement of hygienic handling of camel milk to improve camel milk safety (Adugna et al., 2015, Wasie et al., 2015).

Twelve studies sampled raw cow milk (n = 1293) and reported a median prevalence of *Salmonella* spp. of 6%, with prevalence ranging from 0 to 44% (Abunna et al., 2017, Abunna et al., 2018a, Abunna et al., 2018b, Banti, 2018, Ejo et al., 2016, Mossie and Dires, 2016, Mulaw, 2017, Reta et al., 2016, Tadesse and Dabassa, 2012, Tesfay et al., 2013) (Fig. 3, Supplementary material Table S1). These results suggest that the microbial quality of milk represents a public health concern, since the majority of milk consumed in the country is raw, unpasteurised or un-boiled milk (Amenu et al., 2019, Girma and Abebe, 2018). In addition, milk is commonly promoted by development agencies as a nutritious food for children, thus nutrition and food safety strategies result in common target populations. Identification of source attribution for contamination of raw milk with *Salmonella* spp. could inform future pathogen control strategies aimed at reducing the contamination.

In addition to raw milk, other studies investigated the presence of *Salmonella* spp. in dairy products, including butter, cheese and yogurt (Tesfaw et al., 2013), cottage cheese and cream cakes (a cake with dairy cream-based filling; Ejo et al., 2016), milk for which the pasteurisation status was not reported, pasteurised milk (Tesfaw et al., 2013). Tesfaw et al. (2013) aimed to identify the prevalence of *Salmonella* in butter, cheese, milk and yogurt available in retail stores in Addis Ababa. They had reported a 1% prevalence of *Salmonella* spp. in butter, 3% prevalence in cheese, and 2% prevalence in milk. None of the samples tested in Tesfaw et al. (2013) study were positive for *Salmonella* spp. The Tesfaw et al. (2013) study did not indicate whether the collected samples of milk were raw or pasteurised, nor whether or not the milk was cow milk, thus the prevalence data from this study were not reported in conjunction with data from other studies presented in Fig. 3. Ejo et al. (2016) aimed to identify the prevalence of *Salmonella* spp. in foods from animal origin, including cream cheese and cream cakes in retail stores in Gondar. None of the samples analysed in this study was reported as positive for *Salmonella*. Similarly, Tesfay et al. (2013) tested two pasteurised milk samples and did not detect *Salmonella.* However, due to the small number of samples, these results were not included in the summary shown in Fig. 3.

Based on the results of the reviewed prevalence studies, *Salmonella* spp. represents a health risk in raw milk marketed and consumed in Ethiopia. Further studies are needed to identify the prevalence of this pathogen in pasteurised milk to identify the sources of post-pasteurisation contamination and effectively control the prevalence of this pathogen in the dairy supply chain, considering possible post-pasteurisation contamination. Furthermore, the assessment of interventions geared towards the improvement of sanitary conditions during milking and storage of raw milk are needed.

### 3.4 Prevalence of *Listeria* spp. and *L. monocytogenes* in Ethiopian milk and dairy products

*L. monocytogenes* is a Gram-positive, rod-shaped, facultative anaerobic bacterium that can cause listeriosis in humans. In the USA, foodborne *L. monocytogenes* infections are associated with a 94% hospitalisation rate and 15.9% death rate (Scallan et al., 2011); however, the estimates for Ethiopia are unknown and may differ. Among exposed population, the children, pregnant women, elderly and immunocompromised individuals are at increased risk for listeriosis (Scallan et al., 2011). When epidemiological traceback investigations were conducted on listeriosis-related outbreaks in the USA, *L. monocytogenes* has been linked to dairy foods, including raw milk, ice cream and soft cheese (CDC, 2019). Contamination, including post-pasteurisation contamination with *L. monocytogenes* can be problematic in food processing environments that do not apply GMPs. Since *Listeria* is able to survive and/or grow in diverse environments, including the environments with low temperatures, and foods with high salt concentration and a wide range of pH, environmental pathogen control is an important control measure (Ferreira, Wiedmann, Texeira, & Stasiewicz, 2014). In food processing facilities*, Listeria* spp. may form biofilms in areas that are difficult to clean and sanitise, which can result in recurrent contamination of food products (Colagiorgi et al., 2017).

In Ethiopia, limited research on the prevalence of *L. monocytogenes* in milk and dairy products has been published to date. We identified and reviewed nine peer-reviewed publication on this topic (Derra et al., 2013, Fisseha, 2017, Garedew et al., 2015, Gebretsadik et al., 2011, Girma and Abebe, 2018, Mengesha et al., 2009, Molla et al., 2004, Muhammed et al., 2013, Seyoum et al., 2015). In addition, one non-peer-reviewed doctoral thesis (Desta, 2013) reporting the prevalence of this pathogen within the dairy products was identified but not included in the data analyses and summary reported here. Table 2 presents a summary of the experimental designs used in each of the reviewed studies that had reported the prevalence of *Listeria* spp. and *L. monocytogenes* in different dairy products. All studies involved the collection of samples from retail stores within the urban and peri-urban regions (Fig. 2). Five studies were conducted in Addis Ababa (Derra et al., 2013, Gebretsadik et al., 2011, Mengesha et al., 2009; Molla et al., 2004, Seyoum et al., 2015); two studies were carried out in peri-urban regions up to 200 km from Addis Ababa (Fisseha, 2017, Girma and Abebe, 2018), and two studies were performed in towns over 200 km from Addis Ababa (Garedew et al., 2015, Muhammed et al., 2013) (Fig. 2). Seven studies reported performing a cross-sectional study (Derra et al., 2013, Fisseha, 2017, Garedew et al., 2015, Gebretsadik et al., 2011, Girma and Abebe, 2018; Molla et al., 2004, Muhammed et al., 2013), and four reported a sample size calculation based on the estimated prevalence ranging from 13 to 50% (Fisseha, 2017, Garedew et al., 2015, Girma and Abebe, 2018, Muhammed et al., 2013). The protocols used for enrichment of *Listeria* spp. varied among studies. Six studies employed a protocol with a primary enrichment and secondary enrichment, followed by isolation in solid media (Derra et al., 2013, Garedew et al., 2015, Gebretsadik et al., 2011, Girma and Abebe, 2018, Mengesha et al., 2009; Molla et al., 2004). Two studies used one enrichment step followed by isolation in solid media (Fisseha, 2017, Seyoum et al., 2015). One study diluted the samples in water, before proceeding to the enrichment step (Muhammed et al., 2013). This deviation from standard protocols for detection of *Listeria* spp., may have decreased the rate of *Listeria* recovery. Following isolation, the species confirmation for *L. monocytogenes* was carried out using standard biochemical tests, including Gram staining, haemolysis test, CAMP (Christie-Atkinson-Munch-Peterson) test and sugar fermentation assays (Fisseha, 2017, Garedew et al., 2015, Gebretsadik et al., 2011, Girma and Abebe, 2018, Mengesha et al., 2009; Molla et al., 2004, Muhammed et al., 2013, Seyoum et al., 2015). One study included PCR as a confirmation method, followed by Pulsed-Field Gel Electrophoresis (PFGE) subtyping (Derra et al., 2013).

**Table 2:** Experimental design and locations of reviewed studies that reported the prevalence of *Listeria* spp. and *L. monocytogenes* in Ethiopian dairy products.^a^

Reference	Region	Town	Dates	Place of sampling	Study design	Sample size calculation	Detection method	Confirmation method
Molla et al. (2004)	Addis Ababa	Addis Ababa	Sep 2003 to Apr 2004	Purchased from retail supermarkets and other stores	Cross-sectional	Not stated	Primary enrichment in half Fraser broth, secondary enrichment in Fraser broth, isolation on PALCAM	Gram staining, motility, haemolysis, catalase, CAMP tests, rhamnose, xylose and mannitol fermentation
Mengesha et al. (2009)	Addis Ababa	Addis Ababa	Sep 2004 to Mar 2005	Purchased from 37 supermarkets and pastry stores	Not stated	Not stated	Primary enrichment in half Fraser broth, secondary enrichment in Fraser broth, isolation on PALCAM and Oxford	Haemolysis, CAMP test, rhamnose, xylose and mannitol fermentation, serotyping
Gebretsadik et al. (2011)	Addis Ababa	Addis Ababa	Nov 2008 to Mar 2009	Purchased form retail shops, cafeterias and markets	Cross-sectional	Random sampling; no sample size calculation reported	Pre-enrichment in BLEB, primary enrichment in UVM I and secondary enrichment in UVM II broth, isolation on PALCAM	Gram staining, motility, haemolysis, catalase, CAMP tests, rhamnose, xylose and mannitol fermentation
Derra et al. (2013)	Addis Ababa	Addis Ababa	Jul to Dec 2006	Purchased at retail markets, shops, supermarkets and food vendors	Cross-sectional	Not stated	IDF, 1990	PCR and PFGE
Muhammed et al. (2013)	Oromia	Jimma	Oct 2010 to Mar 2011	Purchased at cafes, milk open markets and pastries.	Cross-sectional	Random sampling. Sample size determined based on P = 13%, d = 5% and confidence = 95%	Sample dilution 1:10 in buffered peptone water. Enrichment in *Listeria* enrichment broth, isolation on Oxford	Gram staining, motility, haemolysis, catalase, CAMP tests, rhamnose, xylose and mannitol fermentation
Garedew et al. (2015)	Amhara	Gondar	Nov 2012 to Jun 2013	Purchased from randomly selected dinning houses (cafeterias, hotels, restaurants and retail shops)	Cross-sectional	For all products combined. Sample size determined based on P = 50%, d = 5%, confidence = 95%.	Primary enrichment in half Fraser broth, secondary enrichment in Fraser broth, isolation on PALCAM	Gram staining, motility, haemolysis, catalase, CAMP tests, rhamnose, xylose and mannitol fermentation
Seyoum et al. (2015)	Addis Ababa	Addis Ababa	Nov 2011 to Jul 2012	Purchased from supermarkets	Not stated	Not stated	Enrichment in LEB broth, isolation on Oxford agar	Catalase, xylose, mannitol and rhamnose fermentation, CAMP test
Fisseha (2017)	Oromia	Bishoftu and Dukem	Nov 2016 to Apr 2017	Restaurants, milk collection centres, supermarkets, cafeterias and small ice cream shops	Cross-sectional	Sample size determined based on P = 32.6%, d = 5%, confidence = 95%.	Enrichment in LEB broth, isolation on PALCAM and OXA agar	Gram staining, motility, haemolysis, catalase, CAMP tests, rhamnose, xylose and mannitol fermentation
Girma and Abebe (2018)	Amhara	Debre-birhan	Not mentioned	Dairy producers that deliver their milk to collection centres	Cross-sectional	Sample size determined based on P = 50%, d = 5%, confidence = 95%	Primary enrichment in half Fraser broth, secondary enrichment in Fraser broth, isolation on Oxford	Gram staining, motility, haemolysis, catalase, CAMP tests, rhamnose, xylose and mannitol fermentation

a Abbreviations: d, precision; P, prevalence; CAMP test, Christie-Atkins-Munch-Peterson test; LEB, listeria enrichment broth; OXA, Oxford agar; PALCAM, polymyxin acriflavine lithium chloride ceftazidime aesculin mannitol agar; PCR, polymerase chain reaction; PFGE, pulsed-field gel electrophoresis; UVM, University of Vermont broth.

**Fig. 4:**
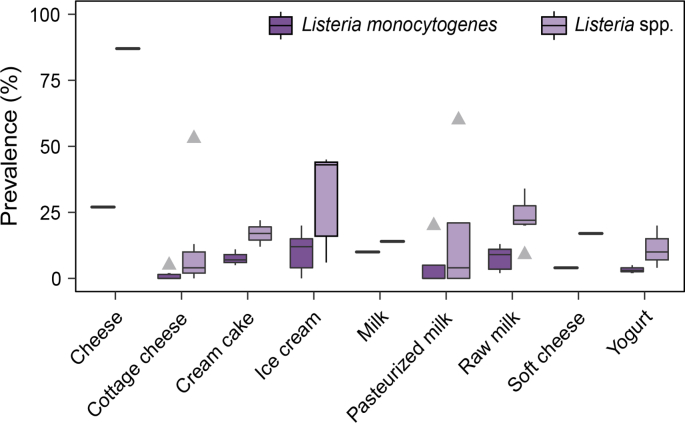
Prevalence of *Listeria* spp. and *L. monocytogenes* in milk and dairy products collected in Ethiopia. N = number of studies, n = number of samples: cheese (N = 1, n = 15), cottage cheese (N = 7, n = 431), cream cake (N = 3, n = 232), ice cream (N = 5, n = 244), milk (N = 1, n = 50), pasteurised milk (N = 3, n = 125), raw milk (N = 6, n = 1040), soft cheese (N = 1, n = 101), yogurt (N = 3, n = 100).

Six studies sampled raw cow milk (n = 1040) and reported a median prevalence of 22%, ranging from 9 to 34% for *Listeria* spp. and a median prevalence of 9%, ranging from 2 to 13% for *L. monocytogenes* (Derra et al., 2013, Fisseha, 2017, Garedew et al., 2015, Gebretsadik et al., 2011, Girma and Abebe, 2018, Seyoum et al., 2015) (Fig. 4, Supplementary material Table S1). These results represent a public health concern, since milk is commonly consumed raw in Ethiopia (Amenu et al., 2019, Tadesse, 2018). High prevalence of *L. monocytogenes* in raw milk is particularly concerning among communities that primarily feed raw milk to children, since listeriosis is of concern among susceptible populations, including infants, the elderly, pregnant women and the immunocompromised (Scallan et al., 2011).

Four studies sampled pasteurised milk (n = 190) and reported a median prevalence of 4%, ranging from 0 to 60% for *Listeria* spp. and a median prevalence of 0%, ranging from 0 to 20% for *L. monocytogenes* (Fisseha, 2017, Garedew et al., 2015, Mengesha et al., 2009; Seyoum et al., 2015) (Fig. 4, Supplementary material Table S1). Seyoum et al. (2015) reported a 20% prevalence of *L. monocytogenes* among 65 tested units of pasteurised milk collected in Addis Ababa. *L. monocytogenes* does not survive pasteurisation (Piyasena, Liou, & McKellar, 1998), which indicates that the high prevalence is likely a result of post-processing contamination or pasteurisation process failure (e.g., insufficient time at a target hold temperature).

Five studies collected and tested samples of ice cream (n = 244) and reported a median prevalence of 43%, ranging from 6 to 45% for *Listeria* spp., and a median prevalence of 12%, ranging from 0 to 20% for *L. monocytogenes* (Fisseha, 2017, Garedew et al., 2015, Mengesha et al., 2009; Molla et al., 2004, Muhammed et al., 2013) (Fig. 4, Supplementary material Table S1). None of the studies reported the sampling strategy for ice cream, including whether the ice cream samples were collected from pre-packaged containers or were dispensed at the retail. This information could provide insight into the potential sources of contamination, which could inform practices aimed at reducing the prevalence of *L. monocytogenes* in the ice cream supply chain. While *L. monocytogenes* is not able to grow at ice cream recommended storage temperature, it can survive freezing temperatures (Salazar, Stewart, Shazer, & Tortorello, 2019) and presents a food safety risk. This has been demonstrated by outbreaks of *L. monocytogenes* in the USA that had been traced back to ice cream as a source of contamination (Chen et al., 2016, Rietberg et al., 2016).

In addition to milk, the prevalence of *L. monocytogenes* was reported in products such as cream cake, cottage cheese, and yogurt. Three studies collected and tested samples of cream cakes (n = 232), and reported a median prevalence of 17%, ranging from 12 to 22% for *Listeria* spp., and a median prevalence of 7%, ranging from 5 to 11% for *L. monocytogenes* (Derra et al., 2013, Garedew et al., 2015, Mengesha et al., 2009) (Fig. 4, Supplementary material Table S1). Cream cakes are usually purchased from bakeries and consumed without further treatment. The results from this study indicate that cream cakes are a source of pathogens that may present a health risk concern, especially for the populations at risk.

Milk produced in Ethiopia is sometimes processed into cottage cheese, also called ayib*.* Ayib is produced by heating the buttermilk, obtained after churning sour milk, to precipitate the protein fraction (Gonfa, Foster, & Holzapfel, 2001). Although ayib has a pH of approximately 3.7, it has a short shelf life due to its high moisture content (Yilma, 2012). Soft cheeses are high moisture content foods that are ripened over a very short time, which does not allow for pathogen die-off. Soft cheeses have intrinsic factors that support microbial growth and they have been implicated in outbreaks of listeriosis in the past (Acciari et al., 2016, Jackson et al., 2011). We identified seven studies that examined the occurrence of *Listeria* in cottage cheese samples (n = 431) in Ethiopia. These studies reported a median prevalence of 4% with prevalence ranging from 0 to 53% for *Listeria* spp., and a median prevalence of 0% with prevalence ranging from 0 to 5% for *L. monocytogenes* (Derra et al., 2013, Fisseha, 2017, Garedew et al., 2015, Gebretsadik et al., 2011, Mengesha et al., 2009, Molla et al., 2004, Muhammed et al., 2013) (Fig. 4, Supplementary material Table S1).

Another dairy product that has been studied, in terms of microbial food safety, is yogurt. Ethiopian traditional yogurt, called ergo, is produced by natural fermentation of raw milk at room temperature in smoked vessels (Gonfa et al., 2001, Yilma, 2012). A study by Ashenafi (1994) showed that *L. monocytogenes* was inactivated when pH of milk fermented into ergo decreased below 3.9. However, the reduction of pH to a level that inactivated *L. monocytogenes* resulted in an unacceptable sensory quality due to the overly sour taste (Ashenafi, 1994). The practice of smoking containers is thought to inhibit spoilage and pathogenic organisms (Yilma, 2012), however further studies are needed to assess the effectiveness of using smoke vessels as a foodborne pathogen control strategy. We included and summarised three studies that collected and tested samples of yogurt (n = 100) in Ethiopia. They had reported a median prevalence of 10%, ranging from 4 to 20% for *Listeria* spp., and a median prevalence of 3% with prevalence ranging from 2 to 5% for *L. monocytogenes* (Fisseha, 2017, Muhammed et al., 2013, Seyoum et al., 2015) (Fig. 4, Supplementary material Table S1). These studies, however, did not report the types of yogurts purchased, their packaging, nor the pH of tested samples.

Additional studies have been identified through the literature review; however, they did not allow for clear classification the tested products into categories outlined above. For example, in a small study, Seyoum et al. (2015) analysed cheese (n = 15) of unidentified type. They reported 87% prevalence of *Listeria* spp. and 27% prevalence of *L. monocytogenes* among tested samples. Furthermore, Mengesha et al. (2009) collected and tested soft cheese samples (possibly cottage cheeses, although authors did not specify the type) (n = 101), and reported a prevalence of 17% for *Listeria* spp. and 4% for *L. monocytogenes*. Lastly, Muhammed et al. (2013) analysed milk samples (n = 50) but did not report whether samples were raw or pasteurised. They reported a 14% prevalence for *Listeria* spp. and 10% prevalence for *L. monocytogenes*.

Overall, above-reported studies show that *L. monocytogenes* represents a health risk related to the consumption of milk and dairy products in Ethiopia. It must be noted that all studies were conducted by purchasing samples from retail centres in peri-urban and urban areas, thus being representative of only a small fraction (∼2%) of milk produced in the country, according to the estimates outlined in the introduction of this review. Hence, further studies focussing on the assessment of the prevalence of *L. monocytogenes* in dairy food samples collected from small-holder farms in rural areas to represent safety of the dairy foods from a predominant market are needed. Inclusion of additional metadata such as specific type of product (e.g., type of yogurt) and location of sampling (e.g., whether sampled ice cream was prepackaged or scooped at retailer) in future studies aiming to determine the prevalence of *L. monocytogenes* in foods would facilitate identification of sources of contamination and critical control points.

### 3.5 Prevalence of *E. coli* O57:H7 in Ethiopian milk and dairy products

*E. coli* is a natural inhabitant of the gastrointestinal tract of mammals (Kaper, Nataro, & Mobley, 2004). While most strains of *E. coli* do not cause disease in humans, some are known to cause severe illness due to the production of toxins and/or other virulence factors (Kaper et al., 2004, Rahal et al., 2012). One of the most concerning pathotypes of *E. coli* is Shiga-toxin producing *E. coli* (STEC or VTEC). A subset of STEC strains can cause a frequently fatal entero-haemorrhagic colitis (HC) and the haemolytic uremic syndrome (HUS) (Robins-Browne et al., 2016). STEC strains that are able to cause HC or HUS are classified as enterohaemorrhagic *E. coli* (EHEC). Among STEC, serotype O157:H7 is well-known to cause foodborne illness, although this serotype is just one of the “big six” STEC serotypes associated with foodborne illness (Kiel et al., 2018). The infectious dose of *E. coli* O157:H7 can be as little as 10–100 cells in susceptible individuals (Kaper et al., 2004, Rahal et al., 2012). Cattle are a known reservoir of this pathogen, and several outbreaks of *E. coli* O157:H7 have been associated with consumption of dairy products (Honish et al., 2004, Keene et al., 1997).

In Ethiopia, research related to the presence of *E. coli* O157:H7 has been mostly focused on slaughterhouses and animal abattoirs (Abdissa et al., 2017, Abreham et al., 2019, Bekele et al., 2014). A recent meta-analysis of the prevalence of *E. coli* O157:H7 in raw animal products in Ethiopia included only one study that collected and analysed milk samples (Assefa, 2019). In this review, five peer-reviewed studies (Abunna et al., 2018c, Adugna et al., 2013, Bedasa et al., 2018, Disassa et al., 2017, Mekuria and Beyene, 2014) were identified and included in the analysis. Additionally, one non-peer reviewed masters' thesis (Hunduma, 2018) was identified but not included in the data analyses and summary in this review. Two studies were conducted in the Oromia region (Abunna et al., 2018c, Bedasa et al., 2018), one in Somali and Dire Dawa regions (Adugna et al., 2013), one in Benishangul-Gumuz region (Disassa et al., 2017), and one in Tigray (Mekuria & Beyene, 2014) (see Fig. 2). Four publications reported conducting a cross-sectional study (Abunna et al., 2018c, Bedasa et al., 2018, Disassa et al., 2017, Mekuria and Beyene, 2014), and two of these four studies reported a sample size calculation based on the expected prevalence of 7.47–50% (Disassa et al., 2017, Mekuria and Beyene, 2014). Table 3 shows a summary of the experimental designs used in these studies and the methods used for isolation and identification of *E. coli* O157:H7 in milk and dairy products. The methods used for isolation and identification of *E. coli* O157:H7 were variable across studies. One of these reviewed studies reported using the standardised method established by ISO (2001) (Bedasa et al., 2018). Two studies isolated and confirmed *E. coli*, and later transferred the colonies to sorbitol McConkey agar to identify putative *E. coli* O157:H7 isolates, which were then confirmed via agglutination test (Abunna et al., 2018c, Disassa et al., 2017). Another study isolated colonies on McConkey agar and later performed confirmation via sugar fermentation tests (including sorbitol) and indole test (Adugna et al., 2013). Finally, one study used a Biolog Identification System to characterise and confirm *E. coli* O157:H7 isolated using Biolog Universal Growth medium (BUG) (Mekuria & Beyene, 2014).

**Table 3:** Experimental design and locations of reviewed studies that reported the prevalence of *E. coli* O157:H7 in Ethiopian dairy products.^a^

Reference	Region	Town	Dates	Place of sampling	Study design	Sample size calculation	Detection method	Confirmation method
Mekuria and Beyene (2014)	Tigray	Mekele, Alamata and Adigrat	Nov-2012 to Jun-2013	Collected from the udders of lactating animals and from the distribution points.	Cross-sectional	Sample size determined based on P = 50%	Streak on blood agar, transfer to BUG	Biolog identification system
Adugna et al. (2015)	Somali and Dire Dawa	Erer and Dire Dawa	Not stated	Collected from producers' households and markets	Not stated	Not stated	Spread plate in MacConkey agar	Sorbitol, arabinose and mannitol fermentation and indole test
Disassa et al. (2017)	Benish-angul-Gumuz	Asosa	Oct-2014 to Jul 2015	Obtained from dairy farmers and milk vendors	Cross-sectional	Sample size determined based on P = 44.4%	Enrichment in EC broth, transfer to EMB agar, isolation on sorbitol McConkey agar	Agglutination test for O157 and H7 antigens
Abunna et al. (2018c)	Oromia	Asella	Nov-2016 to Apr-2017	Collected from farmers, vendors, milk collection centres, markets and kiosks.	Cross-sectional	Not stated	Streak on McConkey agar, transfer to EMB, confirmation for *E. coli* then transfer to sorbitol McConkey	Agglutination test for O157 and H7 antigens
Bedasa et al. (2018)	Oromia	Bishoftu	Nov-2016 to Apr-2017	Obtained from restaurants, open markets and supermarkets.	Cross-sectional	Not stated	ISO 16654:2001	Subculture of confirmed *E. coli* isolates in sorbitol MacConkey agar and agglutination test for O157 and H7 antigens

a Abbreviations: P, prevalence; BUG, Biolog Universal Growth agar; EC, *Escherichia coli* broth; EMB, eosin methylene blue agar.

**Fig. 5:**
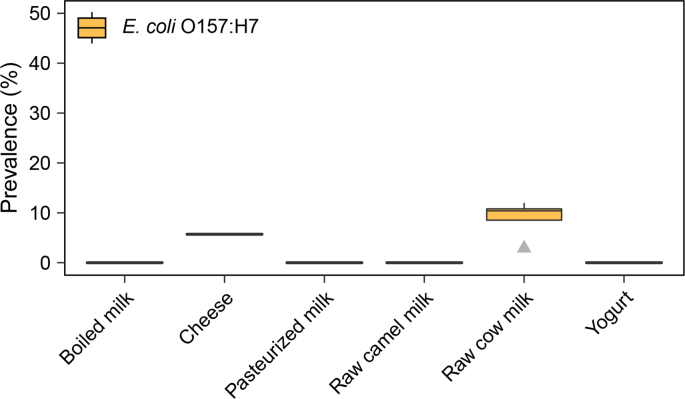
Prevalence of *E. coli* O157:H7 in dairy products collected in Ethiopia. N = number of studies, n = number of samples: boiled milk (N = 1, n = 16), cheese (N = 1, n = 35), pasteurised milk (N = 1, n = 40), raw camel milk (N = 1, n = 24), raw cow milk (N = 4, n = 693), yogurt (N = 1, n = 35).

In one of the above-outlined studies, raw camel milk (n = 24) was sampled and tested, and no *E. coli* O157:H7 was detected in tested samples (Adugna et al., 2013) (Fig. 5, Supplementary material Table S1). Four studies sampled raw cow milk (n = 693) and reported a median prevalence of 10%, ranging from 3 to 12% for *E.coli* O157:H7 (Abunna et al., 2018c, Bedasa et al., 2018, Disassa et al., 2017, Mekuria and Beyene, 2014) (Fig. 5, Supplementary material Table S1).

Several other studies aimed to identify *E. coli* O157:H7 in other dairy products. A small study of Abunna et al. (2018c) collected and tested samples of boiled milk (n = 16) from restaurants and kiosks in Asella, and reported no positive samples. Another small study by Bedasa et al. (2018) sampled and tested cheese (n = 35) and reported 6% prevalence of *E. coli* O157:H7. Lastly, Bedasa et al. (2018) tested pasteurised milk (n = 40) and yogurt (n = 35) and did not detect *E. coli* O157:H7 in any of them.

### 3.6 Prevalence of *Campylobacter* spp. in Ethiopian milk and milk products

*Campylobacter* spp. is a frequent cause of foodborne illness worldwide (Burnham & Hendrixson, 2018). *Campylobacter* spp. are Gram-negative, screw-shaped, microaerophilic bacterium. Among the genus Campylobacter, *Campylobacter jejuni* and *C. coli* are the most prevalent species (Kaakoush, Castaño-Rodríguez, Mitchell, & Man, 2015). Campylobacteriosis symptoms can range from mild diarrhoea to bloody diarrhoea, and *Campylobacter* infections can lead to long-term detrimental neurological consequences in a form of post-infection Guillain-Barré syndrome (Kaakoush et al., 2015). While campylobacteriosis cases have often been linked to consumption of undercooked meat, in particular of poultry, raw milk has also been identified as a source of infection (Davis et al., 2016, Heuvelink et al., 2009, Longenberger et al., 2013, Schildt et al., 2006). Contamination of raw milk with *Campylobacter* is thought to result from faecal contamination during milking (Bianchini et al., 2014, Olivier et al., 2005). In Ethiopia, the presence of *Campylobacter* spp. has been studied in meat products (Abamecha et al., 2015, Kassa et al., 2006, Woldemariam et al., 2010); however, we did not identify any studies examining the prevalence of *Campylobacter* spp. in milk and dairy products, indicating a critical need for future studies to focus on establishing a baseline prevalence of *Campylobacter* spp. in a rapidly growing Ethiopian dairy food production.

## 4 Conclusions and future perspectives

We identified 15, 9, 5 and 0 studies that had reported the prevalence of *Salmonella* spp., *Listeria* spp. and *Listeria monocytogenes, E. coli* O157:H7, and *Campylobacter* spp., respectively, in dairy foods. The reviewed studies reported a median prevalence of *Salmonella, L. monocytogenes,* and *E. coli* O157:H7 of 6, 9 and 10% in raw cow milk in Ethiopia, indicating a concerning level of bacterial foodborne pathogens in raw milk, given the common practice of raw milk consumption. Furthermore, studies that tested pasteurised milk detected *L. monocytogenes* in pasteurised milk as well, albeit at a lower median prevalence across the reviewed studies. This demonstrates the need for investment into food safety development in Ethiopia (e.g., investment in infrastructure and intervention studies) to improve domestic public health and enhance opportunities for Ethiopian participation in international trade (Birke & Zawide, 2019). Combating high prevalence of bacterial foodborne pathogens in raw milk through implementation of effective pathogen control intervention is particularly important due to the fact that raw milk consumption, which is a common practice in Ethiopia, increases the exposure of vulnerable populations (including children) to foodborne pathogens. Overall, the assessment of the effectiveness of educational interventions targeting the reduction of contamination in the dairy supply chain through improvement of knowledge and implementation of good hygiene and production practices is needed to inform larger coordinated efforts focused on improvement of dairy food safety in Ethiopia. Lastly, special culturally sensitive interventions may need to be developed for pastoralist communities to increase the awareness of food safety hazards associated with consumption of raw milk and provide recommendations on how to mitigate these risks through behavioural changes.
