# Supplementary material for: Review of the prevalence of foodborne pathogens in milk and dairy products in Ethiopia
Source: Int Dairy J. 2020 Oct;109:104762. doi: 10.1016/j.idairyj.2020.104762 (PMC7430047; doi:10.1016/j.idairyj.2020.104762)
Supplement: Multimedia component 1 [file mmc1.docx]

**Supplementary material**

**Table S1**

Foodborne pathogen prevalence reported in milk and dairy products collected in Ethiopia.

| Dairy product | n | Organism | Prevalence (%) | Reference |
| --- | --- | --- | --- | --- |
| Butter | 96 | *Salmonella* spp. | 1.04 | Tesfaw et al. (2013) |
| Cheese | 96 | *Salmonella* spp. | 3.1 | Tesfaw et al. (2013) |
| Cottage cheese | 20 | *Salmonella* spp. | 0 | Ejo, Garedew, Alebachew, & Worku (2016) |
| Cream cake | 50 | *Salmonella* spp. | 0 | Ejo, Garedew, Alebachew, & Worku (2016) |
| Milk | 96 | *Salmonella* spp. | 2.1 | Tesfaw et al. (2013) |
| Raw camel milk | 12^b^ | *Salmonella* spp. | 66.4 | Adugna, Seifu, Kebeded, & Doluschitz (2013) |
|  | 12^c^ | *Salmonella* spp. | 92 |  |
|  | 47^a^ | *Salmonella* spp. | 6.38 | Abera, Legesse, Mummed, & Urga (2016) |
|  | 22^b^ | *Salmonella* spp. | 18.18 |  |
|  | 57^c^ | *Salmonella* spp. | 21.05 |  |
| Raw cow milk | 195 | *Salmonella* spp. | 3.07 | Addis et al. (2011) |
|  | 100 | *Salmonella* spp. | 20 | Tadesse & Dabassa (2012) |
|  | 18 | *Salmonella* spp. | 44.44 | Tesfay, Kebede, & Seifu (2013) |
|  | 100 | *Salmonella* spp. | 16 | Abate, Rakshit, & Anal (2013) |
|  | 50 | *Salmonella* spp. | 6 | Ejo, Garedew, Alebachew, & Worku (2016) |
|  | 106 | *Salmonella* spp. | 23.6 | Mossie & Dires (2016) |
|  | 120 | *Salmonella* spp. | 3.3 | Reta, Bereda, & Alemu (2016) |
|  | 91^a^ | *Salmonella* spp. | 12.1 | Abunna et al. (2017) |
|  | 21^d^ | *Salmonella* spp. | 4 |  |
|  | 384 | *Salmonella* spp. | 9.35 | Mulaw (2017) |
|  | 114^a^ | *Salmonella* spp. | 7.02 | Abunna et al. (2018a) |
|  | 19^d^ | *Salmonella* spp. | 0 |  |
|  | 6^d^ | *Salmonella* spp. | 0 | Abunna et al. (2018b) |
|  | 36^a^ | *Salmonella* spp. | 0 |  |
|  | 33 | *Salmonella* spp. | 0 | Banti (2018) |
| Yoghurt | 96 | *Salmonella* spp. | 0 | Tesfaw et al. (2013) |
| Cheese | 15 | *Listeria* spp. | 86.7 | Seyoum, Woldetsadik, Mekonen, Gezahegn, & Gebreyes (2015) |
|  |  | *L. monocytogenes* | 26.7 |  |
| Cottage cheese | 61 | *Listeria* spp. | 1.6 | Molla, Yilma, & Alemayehu (2004) |
|  |  | *L. monocytogenes* | 0 |  |
|  | 80 | *Listeria* spp. | 0 | Mengesha et al. (2009) |
|  |  | *L. monocytogenes* | 0 |  |
|  | 100 | *Listeria* spp. | 4 | Gebretsadik, Kassa, Alemayehu, Huruy, & Kebede (2011) |
|  |  | *L. monocytogenes* | 1 |  |
|  | 60 | *Listeria* spp. | 6.8 | Derra et al. (2013) |
|  |  | *L. monocytogenes* | 1.7 |  |
|  | 50 | *Listeria* spp. | 2 | Muhammed, Muleta, Deneke, Gashaw, & Bitew (2013) |
|  |  | *L. monocytogenes* | 0 |  |
|  | 40 | *Listeria* spp. | 12.5 | Garedew et al. (2015) |
|  |  | *L. monocytogenes* | 0 |  |
|  | 40 | *Listeria* spp. | 52.5 | Fisseha (2017) |
|  |  | *L. monocytogenes* | 5 |  |
| Cream cake | 107 | *Listeria* spp. | 12.1 | Mengesha et al. (2009) |
|  |  | *L. monocytogenes* | 6.5 |  |
|  | 60 | *Listeria* spp. | 22 | Derra et al. (2013) |
|  |  | *L. monocytogenes* | 5.1 |  |
|  | 65 | *Listeria* spp.*.* | 16.9 | Garedew et al. (2015) |
|  |  | *L. monocytogenes* | 10.7 |  |
| Ice cream | 46 | *Listeria* spp. | 43.5 | Molla, Yilma, & Alemayehu (2004) |
|  |  | *L. monocytogenes* | 19.6 |  |
|  | 103 | *Listeria* spp. | 42.7 | Mengesha et al. (2009) |
|  |  | *L. monocytogenes* | 11.7 |  |
|  | 50 | *Listeria* spp. | 6 | Muhammed, Muleta, Deneke, Gashaw, & Bitew (2013) |
|  |  | *L. monocytogenes* | 4 |  |
|  | 20 | *Listeria* spp. | 45 | Garedew et al. (2015) |
|  |  | *L. monocytogenes* | 15 |  |
|  | 25 | *Listeria* spp. | 16 | Fisseha (2017) |
|  |  | *L. monocytogenes* | 0 |  |
| Milk | 50 | *Listeria* spp. | 14 | Muhammed, Muleta, Deneke, Gashaw, & Bitew (2013) |
|  |  | *L. monocytogenes* | 10 |  |
| Pasteurised milk | 50 | *Listeria* spp. | 0 | Mengesha et al. (2009) |
|  |  | *L. monocytogenes* | 0 |  |
|  | 50 | *Listeria* spp. | 0 | Garedew et al. (2015) |
|  |  | *L. monocytogenes* | 0 |  |
|  | 65 | *Listeria* spp. | 60 | Seyoum, Woldetsadik, Mekonen, Gezahegn, & Gebreyes (2015) |
|  |  | *L. monocytogenes* | 20 |  |
|  | 25 | *Listeria* spp. | 8 | Fisseha (2017) |
|  |  | *L. monocytogenes* | 0 |  |
| Raw milk | 100 | *Listeria* spp. | 22 | Gebretsadik, Kassa, Alemayehu, Huruy, & Kebede (2011) |
|  |  | *L. monocytogenes* | 13 |  |
|  | 60 | *Listeria* spp. | 8.5 | Derra et al. (2013) |
|  |  | *L. monocytogenes* | 3.4 |  |
|  | 50 | *Listeria* spp. | 25 | Garedew et al. (2015) |
|  |  | *L. monocytogenes* | 4 |  |
|  | 343 | *Listeria* spp. | 21 | Seyoum, Woldetsadik, Mekonen, Gezahegn, & Gebreyes (2015) |
|  |  | *L. monocytogenes* | 2 |  |
|  | 80 | *Listeria* spp. | 33.7 | Fisseha (2017) |
|  |  | *L. monocytogenes* | 8.8 |  |
|  | 384^i^ | *Listeria* spp. | 20.3 | Girma & Abebe (2018) |
|  |  | *L. monocytogenes* | 8.6 |  |
|  | 23^j^ | *Listeria* spp. | 30.4 |  |
|  |  | *L. monocytogenes* | 13 |  |
| Soft cheese | 101 | *Listeria* spp. | 16.8 | Mengesha et al. (2009) |
|  |  | *L. monocytogenes* | 3.9 |  |
| Yoghurt | 50 | *Listeria* spp. | 4 | Muhammed, Muleta, Deneke, Gashaw, & Bitew (2013) |
|  |  | *L. monocytogenes* | 2 |  |
|  | 20 | *Listeria* spp. | 10 | Seyoum, Woldetsadik, Mekonen, Gezahegn, & Gebreyes (2015) |
|  |  | *L. monocytogenes* | 5 |  |
|  | 30 | *Listeria* spp. | 20 | Fisseha (2017) |
|  |  | *L. monocytogenes* | 3.3 |  |
| Boiled milk | 16 | *E. coli* O157:H7 | 0 | Abunna et al. (2018c) |
| Cheese | 35 | *E. coli* O157:H7 | 5.71 | Bedasa, Shiferaw, Abraha, & Moges (2018) |
| Pasteurised milk | 40 | *E. coli* O157:H7 | 0 | Bedasa, Shiferaw, Abraha, & Moges (2018) |
| Raw camel milk | 12^k^ | *E. coli* O157:H7 | 0 | Adugna et al. (2013) |
|  | 12^l^ | *E. coli* O157:H7 | 0 |  |
| Raw cow milk | 192 | *E. coli* O157:H7 | 10.4 | Mekuria & Beyene (2014) |
|  | 380 | *E. coli* O157:H7 | 2.9 | Disassa, Sibhat, Mengistu, Muktar, & Belina, (2017) |
|  | 96 | *E. coli* O157:H7 | 10.4 | Abunna et al. (2018c) |
|  | 25 | *E. coli* O157:H7 | 12 | Bedasa, Shiferaw, Abraha, & Moges (2018) |
| Yoghurt | 35 | *E. coli* O157:H7 | 0 | Bedasa, Shiferaw, Abraha, & Moges (2018) |

^a^ Collected from the udder of lactating animals.

^b^ Collected from the milking bucket.

^c^ Collected from the market.

^d^ Collected from the bulk tank.

^e^ Collected in Selale.

^f^ Collected in Asella.

^g^ Collected in Debre Zeit.

^h^ Collected in Akaki.

^i^ Raw milk samples were collected from dairy producers.

^j^ Raw milk samples were collected from dairy collection centers.

^k^ Obtained from selected households.

^l^ Obtained from open markets**.**

**References**

Abate, A. A., Rakshit, S. K., & Anal, A. K. (2013). Genotypic and phenotypic characterization of antimicrobial resistance patterns of *Salmonella* strains isolated from raw milk in Sebeta, Ethiopia. *International Journal of Advanced Life Sciences*, *6*, 2277–2758.

Abera, T., Legesse, Y., Mummed, B., & Urga, B. (2016). Bacteriological quality of raw camel milk along the market value chain in Fafen Zone, Ethiopian Somali Regional State. *BMC Research Notes*, *9*, 285.

Abunna, F., Ashenafi, D., Beyene, T., Ayana, D., Mamo, B., & Duguma, R. (2017). Isolation, identification and antimicrobial susceptibility profiles of *Salmonella* isolates from dairy farms in and around Modjo Town, Ethiopia. *Ethiopian Veterinary Journal*, *21*, 92.

Abunna, F., Bedashu, A., Beyene, T., Ayana, D., Wakjira, B., Feyisa, A., et al. (2018a). Occurrence of *Salmonella* and its antimicrobial sensitivity test in abattoir and dairy farms in Adama Town, Oromia, Ethiopia. *Journal of Veterinary Medicine and Research*, *5*, 1127–1134.

Abunna, F., Ngusie, G., Tufa, T. B., Ayana, D., Wakjira, B., Waktole, H., & Duguma, R. (2018b). Occurence and antimicrobial susceptibility profile of *Salmonella* from dairy farms in and around Meki Town, Oromia, Ethiopia. *Biomedical Journal of Scientific & Technical Research*, *6*, 5388–5395.

Abunna, F., Worku, H., Gizaw, F., Ragassa, F., Ayana, D., Amenu, K., et al. (2018c). Assessment of post-harvest handling practices, quality and safety of milk and antimicrobial susceptibility profiles of *Escherichia coli* O157:H7 isolated from milk in and around Asella Town, Oromia, Ethiopia. *Annals of Public Health and Research*, *5*, 1072.

Addis, Z., Kebede, N., Worku, Z., Gezahegn, H., Yirsaw, A., & Kassa, T. (2011). Prevalence and antimicrobial resistance of *Salmonella* isolated from lactating cows and in contact humans in dairy farms of Addis Ababa: A cross sectional study. *BMC Infectious Diseases*, *11*, Article 222.

Adugna, M., Seifu, E., Kebeded, A., & Doluschitz, R. (2013). Quality and safety of camel milk along the value chain in Eastern Ethiopia. *International Journal of Food Studies*, *2*, 150–157.

Banti, H. B. (2018). Isolation, identification and antimicrobial susceptibility profile of *Salmonella* isolates from abattoir and selected dairy farms of Addis Ababa City, Ethiopia. *Global Veterinaria*, *20*, 285–292.

Bedasa, S., Shiferaw, D., Abraha, A., & Moges, T. (2018). Occurrence and antimicrobial susceptibility profile of *Escherichia coli*  O157:H7 from food of animal origin in Bishoftu Town, Central Ethiopia. *International Journal of Food Contamination*, *5*, Article 2.

Derra, F. A., Karlsmose, S., Monga, D. P., Mache, A., Svendsen, C. A., Félix, B., et al. (2013). Occurrence of *Listeria* spp. in retail meat and dairy products in the area of Addis Ababa, Ethiopia. *Foodborne Pathogens and Disease*, *10*, 577–579.

Disassa, N., Sibhat, B., Mengistu, S., Muktar, Y., & Belina, D. (2017). Prevalence and Antimicrobial susceptibility pattern of *E. coli* O157:H7 isolated from traditionally marketed raw cow milk in and around Asosa Town, Western Ethiopia. *Veterinary Medicine International*, *2017*, Article 7581531.

Ejo, M., Garedew, L., Alebachew, Z., & Worku, W. (2016). Prevalence and antimicrobial resistance of *Salmonella* isolated from animal-origin food items in Gondar, Ethiopia. *BioMed Research International*, *2016*, Article 4290506.

Fisseha, S. (2017). Occurence of *Listeria monocytogenes* in ready-to-eat foods of animal origin and its antibiotic susceptibility profile, Bishoftu and Dukem Towns, central Ethiopia. *World Journal of Advance Healthcare Research*, *1*, 47–62.

Garedew, L., Taddese, A., Biru, T., Nigatu, S., Kebede, E., Ejo, M., et al. (2015). Prevalence and antimicrobial susceptibility profile of Listeria species from ready-to-eat foods of animal origin in Gondar Town, Ethiopia. *BMC Microbiology*, *15*, Article 100.

Gebretsadik, S., Kassa, T., Alemayehu, H., Huruy, K., & Kebede, N. (2011). Isolation and characterization of *Listeria monocytogenes* and other Listeria species in foods of animal origin in Addis Ababa, Ethiopia. *Journal of Infection and Public Health*, *4*, 22–29.

Girma, Y., & Abebe, B. (2018). Isolation, identification and antimicrobial susceptibility of Listeria species from raw bovine milk in Debre-Birhan Town, Ethiopia. *Journal of Zoonotic Disease and Public Health*, *2*, 4.

Mekuria, A., & Beyene, T. (2014). Zoonotic bacterial pathogens isolated from food of bovine in selected woredas of Tigray, Ethiopia. *World Applied Sciences Journal*, *31*, 1864–1868.

Mengesha, D., Kleer, J., Gebreyes, W. A., Zewde, B. M., Toquin, M. T., Kleer, J., et al. (2009). Occurrence and distribution of *Listeria monocytogenes* and other *Listeria* species in ready-to-eat and raw meat products. *Berliner Und Munchener Tierarztliche Wochenschrift*, *122*, 20–24.

Molla, B., Yilma, R., & Alemayehu, D. (2004). *Listeria monocytogenes* and other Listeria species in retail meat and milk products in Addis Ababa, Ethiopia. *Ethiopian Journal Health Development*, *18*, 208–212.

Mossie, T., & Dires, A. (2016). Prevalence of antimicrobial resistant *Salmonellae* isolated from bulk milk of dairy cows in and around Debre Zeit, Ethiopia. *World’s Veterinary Journal*, *6*, 110–116.

Muhammed, W., Muleta, D., Deneke, Y., Gashaw, A., & Bitew, M. (2013). Studies on occurance of *Listeria monocytogenes* and other species in milk and milk products in retail market of Jimma Town, Ethiopia. *Asian Journal Dairy & Food Research*, *32*, 35–39.

Mulaw, G. (2017). Prevalence and antimicrobial susceptibility of *Salmonella* species from lactating cows in dairy farm of Bahir Dar Town, Ethiopia. *African Journal of Microbiology Research*, *11*, 1578–1585.

Reta, M. A., Bereda, T. W., & Alemu, A. N. (2016). Bacterial contaminations of raw cow’s milk consumed at Jigjiga City of Somali Regional State, Eastern Ethiopia. *International Journal of Food Contamination*, *3*, Article 4.

Seyoum, E. T., Woldetsadik, D. A., Mekonen, T. K., Gezahegn, H. A., & Gebreyes, W. A. (2015). Prevalence of *Listeria monocytogenes* in raw bovine milk and milk products from Central Highlands of Ethiopia. *Journal of Infection in Developing Countries*, *9*, 1204–1209.

Tadesse, T., & Dabassa, A. (2012). Prevalence and antimicrobial resistance of *Salmonella* isolated from raw milk samples collected from Kersa District, Jimma zone, southwest Ethiopia. *Journal of Medical Sciences*, *12*, 224–228.

Tesfaw, L., Taye, B., Alemu, S., Alemayehu, H., Sisay, Z., & Negussie, H. (2013). Prevalence and antimicrobial resistance profile of *Salmonella*  isolates from dairy products in Addis Ababa, Ethiopia. *African Journal of Microbiology Research*, *7*, 5046–5050.

Tesfay, T., Kebede, A., & Seifu, E. (2013). Quality and safety of cow milk produced and marketed in Dire Dawa Town, Eastern Ethiopia. *International Journal of Integrative Sciences, Innovation and Technology*, *2*, 1–5.
